# Application of fMRI techniques in the study of acupuncture for gynecological diseases: A review

**DOI:** 10.1097/MD.0000000000033268

**Published:** 2023-03-10

**Authors:** Renming Liu, Min He, Mengmeng Sun, Lin Wang, Jiazhen Cao, Qianhui Yu, Fuchun Wang, Tie Li

**Affiliations:** a Acupuncture and Moxibustion Academy, Changchun University of Chinese Medicine, Changchun, China; b Northeast Asian Institute of Traditional Chinese Medicine, Changchun University of Chinese Medicine, Changchun, China.

**Keywords:** acupuncture, fMRI, gynecology, visual techniques

## Abstract

Acupuncture therapy, as a characteristic of Chinese medical therapy, has a long history and remarkable effect in the treatment of gynecological diseases, and so far, it has formed a complete treatment system, but its efficacy and mechanism of action remain unclear. Functional magnetic resonance imaging, a visual technique, provides an objective basis for the study of acupuncture in the treatment of gynecological diseases. This paper summarizes the current status of acupuncture in the treatment of gynecological diseases and summarizes the progress of functional magnetic resonance imaging research related to acupuncture in the treatment of gynecological diseases in the past 10 years, mainly including the common types of gynecological diseases in acupuncture clinics, and the commonly used acupuncture points. This study is expected to provide literature support for subsequent research on the central mechanisms of acupuncture in the treatment of gynecological diseases.

## 1. Introduction

Gynecological diseases, with a large variety and complex mechanisms and a wide age range span, seriously affect people’s quality of life and contribute to the financial burden of society.^[1–4]^ Up to now, the combination of Chinese and Western medicine is still the main treatment option for gynecological diseases,^[5–7]^ but acupuncture therapy is a conventional treatment for some specific gynecological diseases with precise efficacy and significant effects, with a minority of reported adverse effects. Therefore, more and more acupuncture therapies are being performed in gynecological diseases, but the introduction of imaging techniques is essential in order to clarify the main mechanisms by which they exert their efficacy. In recent years, the commonly used imaging techniques include ultrasound, computed tomography imaging, emission computed tomography imaging, magnetic resonance imaging (MRI), and X-ray, but they are not widely used due to their inherent limitations.^[8,9]^ With the development of neuroimaging technology, the emergence of functional MRI (fMRI) has provided a more effective means of studying the mechanisms of acupuncture in the treatment of gynecological diseases. fMRI not only provides real-time imaging of the brain but also has the advantage of noninvasive, high-quality imaging with high spatial resolution compared to other techniques.^[10]^

This technological approach, which can already be investigated in several fields, is easily reproducible and therefore has made some progress and great potential in the field of gynecology. The author investigated the mechanism of acupuncture for gynecological diseases based on the fMRI technique, using the China National Knowledge Infrastructure and PubMed databases as the main databases and combining the keywords: acupuncture, pain, neuroimaging, and selected gynecological disease research directions, and included relevant original studies from 2012 to 2022, for the last 10 years, excluding animal experiments, conference papers, case reports, and protocol studies. The detailed types of gynecological diseases treated by acupuncture and the acupoints taken for the treatment of gynecological diseases are reviewed below for future research.

## 2. Application of fMRI in acupuncture for gynecological diseases

fMRI is an important technique for measuring acupuncture-induced brain activity,^[11,12]^ which can be traced back as far as the 1990s.^[13]^ It works as a blood oxygen level-dependent fMRI technique by monitoring changes in blood oxygen concentration in active brain regions to reflect functional connections between different brain regions.^[14]^ It can effectively combine the 3 factors of function, imaging, and anatomical morphology, with the advantages of high operability, high safety, good stability, and many analysis methods.^[15]^ As an important technological method for evaluating brain function, fMRI has been widely used and studied, and researchers have classified fMRI into resting-state fMRI and task-state fMRI according to different acquisition states.^[16]^ In addition, common analysis methods for fMRI include functional integration analysis as well as functional separation analysis.^[17]^ Therefore, fMRI techniques have higher applicability for studying the mechanisms of acupuncture in the treatment of gynecological diseases.

**Figure 1.**
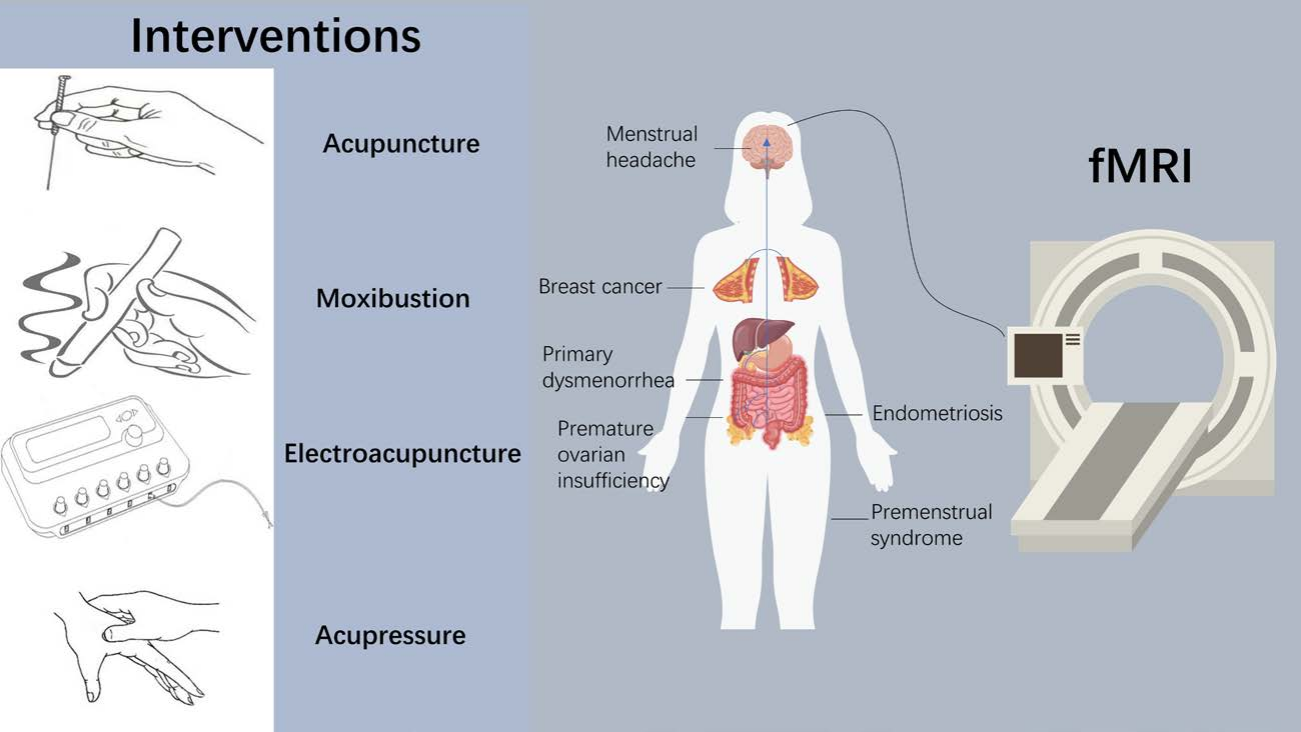
fMRI study of acquisitions for biological diseases. fMRI = functional magnetic resonance imaging.

Menstrual headache is a specific type of migraine, and some scholars have studied the functional brain connectivity of patients based on resting-state fMRI techniques,^[18]^ local-and-distant acupoints method,^[19]^ and physiological mechanisms were studied.^[20]^ It is believed that the precuneus brain function connection is the main pathological factor of menstrual headache and it is also closely related to the regulation of distal acupoints, while the frontal lobe-limbic region is also the main site for acupuncture to regulate menstrual migraine patients.^[21]^ Women with premenstrual syndrome have greater amygdala volumes.^[22]^ Gaoxiong Duan et al studied premenstrual syndrome and abnormal neural activity and found that acupuncture treatment could regulate neural activity in premenstrual syndrome patients, and showed a functional connection between the amygdala and some brain regions.^[23,24]^ Furthermore, many scholars have conducted in-depth studies on primary dysmenorrhea, respectively from the perspective of acupoints and sham acupoints,^[25]^ true or sham acupuncture therapy,^[26]^ brain function connection network,^[27–30]^ disease and health,^[31]^ and heat-sensitive state.^[32]^ They found abnormalities in brain structure and function in patients with primary dysmenorrhea compared to healthy controls,^[33]^ The clinical symptoms performed by patients with primary dysmenorrhea are related to the frequency of abnormal brain function activity, and the nature of their pain is the hyperconnectivity of the primary somatosensory area (S1)/primary motor area around the cerebral aqueduct, the ventral lateral prefrontal lobe, and the posterior cingulate when primary dysmenorrhea patients are in menstruation, among others,^[34]^ and the change in cerebral blood volume is the mechanism that can verify the efficacy of acupuncture.^[35,36]^ Premature uterine dysfunction can lead to a variety of menstrual disorders. Using resting-state fMRI, Tingting Zhao et al compared the functional connectivity of brain regions in patients with premature ovarian dysfunction with that of healthy subjects and explored the central mechanism of acupuncture in the treatment of premature uterine dysfunction, suggesting that the mechanism may be related to the regulation of functional connectivity between the hypothalamus and multiple brain regions.^[37]^ A Swedish research team found that acupuncture therapy is quite an efficacy intervention for reducing serum anti-Mullerian hormone levels.^[38]^ Karin Meissner et al also combined moxibustion with psychotherapy to evaluate the degree of pain relief in patients with endometriosis with this therapy. Interestingly, they found that moxibustion with psychotherapy reduced global pain and pelvic pain.^[39]^ Researchers have shown that even self-administered acupressure could have a good effect on fatigued breast cancer survivors.^[40]^ Details of fMRI in acupuncture therapies for gynecological diseases could be found in Table 1 and Figure 1.

**Table 1:** fMRI in acupuncture therapies for gynecological diseases.

First author and affliction	Diseases	Intervention	Acupoints	Primary results	Year	Reference
Tingting Zhao, Affiliated Hospital of Nanjing University of Chinese Medicine, China	Premature ovarian insufficiency	Electroacupuncture	DU20, RN12, DU24, RN4, ST25, EX-CA1, ST36, SP6, KI12, BG13, LR11, LR3, BL23, BL32	Acupuncture can improve ovarian function and clinical symptoms, and the mechanism may be related to the regulation of functional connections between the hypothalamus and several brain regions.	2022	^[37]^
Tongxiao Ma, Shaanxi University of Chinese Medicine, China	Primary dysmenorrhea	Manual acupuncture	RN4, SP6	Compared to ibuprofen treatment, acupuncture has better efficacy and is expected to fundamentally correct abnormalities in central regulatory pathways for primary dysmenorrhea.	2022	^[31]^
Jun Xiong, Jiangxi University of Chinese Medicine, China	Primary dysmenorrhea	Moxibustion	RN4, EX-CA1, BL32, SP6	Local coherence coordination of brain regions with moxibustion is dominated by positive activation, where the prefrontal lobe may play a key role in analgesic sensation and analgesic mood.	2022	^[29]^
Xingchen Zhou, Jiangxi University of Chinese Medicine, China	Primary dysmenorrhea	Moxibustion	RN4	Moxibustion of RN4 enhances spontaneous neuronal activity in the middle frontal gyrus and precuneus; decreases spontaneous neuronal activity in the cuneus, occipital supraoccipital gyrus, and middle occipital gyrus.	2022	^[30]^
Yutong Zhang Chengdu University of Traditional Chinese Medicine, China	Menstrual migraine	Manual acupuncture	GB20, GB8, PC6, SP6, LR3, NA5	Acupuncture is effective in treating both migraines and can improve emotional symptoms. The mechanism may be through the regulation of mood disorders in frontal-brain regions.	2021	^[20]^
Gaoxiong Duan The People’s Hospital of Guangxi Zhuang Autonomous Region, China	Premenstrual syndrome	Electroacupuncture	SP6	Imaging evidence supports that acupuncture stimulation associated with SP6 modulates neural activity in patients with premenstrual syndrome.	2021	^[24]^
Yong Pang, Guangxi University of Chinese Medicine, China	Premenstrual syndrome	Electroacupuncture	SP6	Stimulation at SP6 increased functional connectivity between the left amygdala and brainstem, right hippocampus, and decreased functional connectivity between the left amygdala and left thalamus, bilateral supplementary motor area.	2021	^[23]^
Chenghao Tu, China Medical University of China, Taiwan	Primary dysmenorrhea	Manual acupuncture	SP6	Acupuncture may block alterations in functional connectivity in the downstream pain modulation system of dysmenorrhea.	2021	^[26]^
Ziwen Wang, Chengdu University of Traditional Chinese Medicine, China	Menstrual migraine	Manual acupuncture	GB20, GB8, LR3, PC6	Distal allotment acupuncture for menstrual migraine showed improvement in the degree of headache and it was suggested that this modulation may be related to the occipital and precuneus lobes.	2021	^[19]^
Yanan Wang, Chengdu University of Traditional Chinese Medicine, China	Primary dysmenorrhea	Manual acupuncture	SP6	A post- versus pretreatment change in the functional connectivity of the rACC and left precentral gyrus in the comparison of real acupuncture versus sham acupuncture.	2021	^[25]^
Yutong Zhang, Chengdu University of Traditional Chinese Medicine, China	Menstrual migraine	Manual acupuncture	GB20, GB8, SP6, PC6, LR3	Acupuncture is effective in treating menstrual migraine. The abnormal precuneus brain functional connectivity in patients with primary dysmenorrhea may be a central pathological factor.	2020	^[18]^
Dingyi Xie, Jiangxi University of Chinese Medicine, China	Primary dysmenorrhea	Moxibustion	RN4	Moxibustion can change the brain’s functional connectivity network. It may enhance and inhibit different brain regions.	2019	^[32]^
Richard E. Harris, University of Michigan, USA	Fatigued breast cancer	Acupressure	DU29, Anmian, HT7, SP6, LR3, CV-6 LI4, ST36, SP6, KI3	There are specific mechanisms of action of self- administered on different acupoints for fatigued breast cancer survivors.	2017	^[40]^
Karin Meissner, Hannover Medical School, Germany	Endometriosis	Psychotherapy with moxibustion	CV3	Moxibustion with psychotherapy reduced overall pain, pelvic pain, and excretion disorders in patients with endometriosis and improved their quality of life.	2016	^[39]^
Chengguo Su, Zhengzhou First People’s Hospital, China	Primary dysmenorrhea	Manual acupuncture	SP6, SP8	The analgesic effect of acupuncture therapy may be achieved through the interaction of multiple functional brain regions.	2016	^[28]^
Henrik Leonhardt, Sahlgrenska Academy, Sweden	Polycystic ovary syndrome	Electroacupuncture	CV3, CV6, ST29, SP6, SP9, LI4, PC6	Electroacupuncture therapy could reduce serum anti-Mullerian hormone levels and ovarian volume.	2015	^[38]^
Yune Song Chongqing Medical University, China	Primary dysmenorrhea	Moxibustion	RN4	Moxibustion of RN4 could cause functional changes in several brain regions associated with pain.	2012	^[27]^

fMRI = functional magnetic resonance imaging.

## 3. fMRI study of acupoints for gynecological diseases

The concept of holistic and evidence-based practice has always been the 2 most important basic features of the theoretical system of Traditional Chinese medicine, and evidence-based practice is used throughout every disease, every evidence type, and the beginning and end of every symptom. Based on these principles, we summarize the basic points and main points commonly used in gynecological diseases.

In Tradition Chinese gynecology, the uterus point is often used as an extra-menstrual point to treat menstrual disorders, infertility, uterine prolapse, etc. Therefore, in recent years, many research groups have investigated the mechanism of the acupuncture and moxibustion of the uterus point through fMRI techniques and concluded that stimulation of the uterus point induces functional activity in brain regions such as the precuneus, cerebellum, postcentral gyrus, talar sulcus, and lingual gyrus, whose neural activity is closely related to reproductive hormone levels.^[41,42]^ As an important point in the treatment of gynecological diseases, Tai Chong acupoint has been widely used in acupuncture clinics, and some domestic and foreign research groups have used fMRI techniques to explore the brain function mechanism of acupuncture at Tai Chong acupoint and found that acupuncture and moxibustion at Tai Chong acupoint can activate specific brain areas, and the activated brain areas are closely related to their main functions.^[43–46]^ According to traditional Chinese medicine theory, the Sanyinjiao acupoint is the intersection of the 3 meridians, namely, the Liver Meridian, the Spleen Meridian, and the Kidney Meridian, which can tonify the blood and benefit the qi, transport the heart and kidneys, and is used for a variety of gynecological disorders,^[47,48]^ and is often applied in cases related to liver stagnation. Some researchers have found that acupuncture stimulation of the Sanyinjiao in healthy subjects alone resulted in 2 opposite effects of activation and inhibition of specific brain regions, objectively confirming the bidirectional regulatory effects of acupuncture.^[49]^ In addition, the Taixi point, the original acupoint of the kidney meridian, has the function of regulating the punch and nourishing the kidney to nourish the marrow and is often used in the gynecological classification of the kidney deficiency type.^[50]^ A group of researchers found that acupuncture at the Taixi point enhanced brain network links between the hippocampus and caudate nucleus, and the prefrontal cortex and hippocampus.^[51]^ In the clinical application of acupuncture, not only can single acupoints improve specific symptoms, but also can be used to systematically regulate the disease state of the human body.^[52,53]^

## 4. Conclusion

Acupuncture treatment of gynecological diseases is diverse, and the brain areas activated or inhibited are not identical among different groups of subjects in different disease states. Acupuncture can modulate the pathological state of the body, and the brain functional connectivity based on the fMRI technique varies among different types of gynecological diseases, different age groups, and different traditional Chinese medicine evidence groups.^[54–56]^ Based on the fMRI studies, we found the mechanism of treating some diseases by acupuncture therapies and the functional specificity of acupoints. Therefore, these results will help guide stimulation protocols directed at the brain network in the future.

Although animal models can enable the study of intrinsic genetic conditions, extrinsic environmental factors, pathogenesis, and even treatment options without human or in vitro experiments, no animal model can completely replicate a complex human disease because of the fundamental genetic relationship between animals and humans, and each animal model has its own shortcomings. In addition, although fMRI technology can be traced back to 1990, it has only been widely used and systematically tested in recent years, so studies in the last decade are used as the main reference.^[57]^

In the course of our study, we found that primary dysmenorrhea and menstrual headache among gynecological diseases were studied more frequently, and these diseases were studied in detail, with objective and reasonable control groups, which may be related to the hot research on the analgesic mechanism of acupuncture in recent years. In addition, we set the interventions as “acupuncture” and “moxibustion,” but when we summed up and classified the literature, we found that acupuncture is more common than moxibustion as the main intervention, which may be related to the high operability of acupuncture and the high acceptance of patients. Common gynecological conditions usually occur with painful symptoms. Acupuncture analgesia has a good clinical outcome. And the pain relief effect of acupuncture is primarily due to the activation of the nociceptive regulatory system and the limbic system related to nociceptive cognition in the brain which leads to some corresponding effect changes. In addition, fMRI is an imaging method that could study brain function in vivo, with high temporal and spatial resolution, and has made remarkable achievements in recent years in exploring the mechanism of acupuncture therapies, so fMRI has great feasibility in observing acupuncture therapies for gynecological diseases.^[58]^ Furthermore, gynecological diseases are so diverse and cover such a wide range that many types of diseases have not been studied under fMRI techniques, which is most likely related to the operability of their trials. Therefore, in the future, if we want to carry out the application of functional MRI technology in acupuncture for gynecological diseases in a comprehensive and multi-level manner, we should investigate the shortcomings and fill in the gaps, strictly standardize the test steps and details, and effectively combine the visualization technology from the clinical perspective of acupuncture to better serve the patients.

## Author contributions

**Conceptualization:** Renming Liu.

**Data curation:** Min He, Qianhui Yu.

**Formal analysis:** Mengmeng Sun, Jiazhen Cao.

**Funding acquisition:** Min He, Mengmeng Sun, Tie Li.

**Investigation:** Mengmeng Sun, Qianhui Yu.

**Methodology:** Lin Wang, Jiazhen Cao, Qianhui Yu.

**Project administration:** Tie Li.

**Resources:** Renming Liu, Jiazhen Cao.

**Validation:** Min He, Fuchun Wang.

**Visualization:** Min He, Tie Li.

**Writing – original draft:** Renming Liu.

**Writing – review & editing:** Min He, Tie Li.
